# Investigation of Potential Drug Targets Involved in Inflammation Contributing to Alzheimer’s Disease Progression

**DOI:** 10.3390/ph17010137

**Published:** 2024-01-20

**Authors:** Catherine Sharo, Tianhua Zhai, Zuyi Huang

**Affiliations:** Department of Chemical and Biological Engineering, Villanova University, Villanova, PA 19085, USA

**Keywords:** Alzheimer’s disease, inflammation, protein interaction network, drug target, drug discovery, classical complement pathway, G protein-coupled receptors, cytokines

## Abstract

Alzheimer’s disease has become a major public health issue. While extensive research has been conducted in the last few decades, few drugs have been approved by the FDA to treat Alzheimer’s disease. There is still an urgent need for understanding the disease pathogenesis, as well as identifying new drug targets for further drug discovery. Alzheimer’s disease is known to arise from a build-up of amyloid beta (Aβ) plaques as well as tangles of tau proteins. Along similar lines to Alzheimer’s disease, inflammation in the brain is known to stem from the degeneration of tissue and build-up of insoluble materials. A minireview was conducted in this work assessing the genes, proteins, reactions, and pathways that link brain inflammation and Alzheimer’s disease. Existing tools in Systems Biology were implemented to build protein interaction networks, mainly for the classical complement pathway and G protein-coupled receptors (GPCRs), to rank the protein targets according to their interactions. The top 10 protein targets were mainly from the classical complement pathway. With the consideration of existing clinical trials and crystal structures, proteins C5AR1 and GARBG1 were identified as the best targets for further drug discovery, through computational approaches like ligand–protein docking techniques.

## 1. Introduction

Alzheimer’s disease is a neurodegenerative disease associated with damaged neurons in the brain that cause progressive memory loss and impaired functions. There are more than 35 million people worldwide who are impacted by this disease, and cases are arising with an increasing trend [1], with more than 100 million predicted to suffer from the disease by 2040–2050 [2]. Thus, this disease is becoming an increasing concern as it continues to negatively impact not only the patients themselves, but also their families and the healthcare system as a whole [3]. Scans, such as Positron Emission Tomography, to check for selected biomarkers (e.g., amyloid beta plaques, tau pathology, and neurodegeneration) cost around USD 3000 per test and expose patients to high levels of radiation, while cerebrospinal fluid tests are invasive with significant risk [2]. Due to the degenerative nature of the disease, care requirements and costs only increase with time, placing higher demands on the patients and those caring for them. The degeneration begins slowly when the patient has a mild form of the disease, but it accelerates as the disease progresses. However, the change in progression is often abrupt, increasing the difficulty of providing patient care [4]. Initial stages of the disease can begin with a long stretch of being asymptomatic. Diagnostic tools for this period could assist in detecting the presence of the disease before symptoms manifest, but there have not yet been any specific biomarkers established as identifying factors for the disease [5]. Alzheimer’s disease is linked to an accumulation of insoluble amyloid beta (Aβ) plaques and tangles of tau proteins [6]. The level of Aβ plaque buildup has been hypothesized to directly correlate with the progression of Alzheimer’s symptoms, eventually resulting in brain atrophy and death [2]. Therefore, these two abnormalities have become key targets in evaluating how the disease could be mitigated or treated.

Despite decades of research and numerous attempts, very few drugs have been approved by the FDA to help treat Alzheimer’s disease or mitigate the symptoms of Alzheimer’s disease. Three of the approved drugs, donepezil, rivastigmine, and galantamine, are acetylcholinesterase inhibitors that focus on promoting neurotransmission, slowing the progression of symptoms but not stopping the disease [2]. A fourth drug, memantine, is a N-methyl D-aspartate receptor ion channel antagonist that is able to reduce tau levels through the activation of phosphoprotein phosphatase 2A [2,7,8]. Only two drugs have been approved that aim to stop disease progression rather than treat the symptoms. In June of 2021, the drug aducanumab, a monoclonal antibody that targets Aβ plaques, was approved, although it has the undesirable effects of inducing vasogenic edema and cerebral microhemorrhage [2]. Leqembi (also named as lecanemab-irmb), another monoclonal antibody targeting Aβ, was approved in January 2023 for patients in the early stages of dementia [9,10]. Most other drugs targeting Aβ have not successfully progressed through clinical trials, primarily due to their lack of curative efficacy. An example of this was verubecestat, a BACE-1 inhibitor that did not help either moderate or early-stage patients [2]. Other BACE-1 inhibitors, as well as PSEN-1 inhibitors, show severe side effects due to their suppression of additional enzymes that are essential for biological functionality [2]. Moreover, there exists an additional challenge: any medication for treating Alzheimer’s disease needs to be able to cross the blood–brain barrier. Any targets chosen in the brain also need to be checked to see that they are not vital to other pathways or functions. An example of this would be GSK-3β inhibitors, as these targets are necessary for glucose metabolism and Wnt-β-catenin, a signaling pathway involved in tissue homeostasis [2,11]. This means that there is a serious demand for drug treatment options that aim to be curative rather than just treat the symptoms of Alzheimer’s disease. In order to generate the best possible options, key targets for the drugs need to be identified. A starting point may involve looking at what the disease shares comorbidities with, such as inflammation in the brain. Along similar lines to Alzheimer’s disease, inflammation in the brain is caused by a combination of degenerating tissue and a buildup of abnormal insoluble materials [12]. Thus, there is a clear potential link between the pathology of Alzheimer’s disease and inflammation in the brain, especially as the disease progresses to later stages, where neuronal death and degeneration levels are higher. As the Aβ plaques and tau tangles begin forming at the early onset of Alzheimer’s disease, their presence only increases as the disease continues. Therefore, the cytokines that mediate inflammation in the regions where Alzheimer’s disease has affected the brain are chronically upregulated [12]. While it was originally thought that inflammation occurred alongside Alzheimer’s disease due to the dead tissue caused by excess Aβ and tau, the constant presence of inflammation is now hypothesized to increase the neurodegenerative progression of Alzheimer’s disease and attack living neural tissues [12]. The linkage of inflammation to cytokine production leads to behavioral changes known as sickness behavior, involving reduced activity, appetite, and social interaction, as well as fever [13]. These cytokines may also exacerbate neuronal loss due to Aβ plaques and the accumulation of tau protein [13]. 

Originally, it was thought that non-steroidal anti-inflammatory drugs (NSAIDs) would prevail in the treatment of Alzheimer’s disease, or at least slow the progression, but there have been no positive trial results so far [2,13,14]. This may stem from the fact that the mechanisms involved in reducing inflammation in patients with Alzheimer’s disease are currently unclear [13]. However, additional NSAID-derived options are also being explored. CereSpir is investigating a small molecule called CSP-1103 that is made via an NSAID scaffold that has reduced COX-1 inhibition and removed COX-2 inhibition, allowing it to increase microglial phagocytic activity and decrease tissue-damaging cytokines [15,16]. Retinoid X receptors, such as bexarotene (i.e., Targretin), have similarly been investigated, as retinoids play a role in immune responses, including the production of apolipoprotein E (ApoE), induction of microglial phagocytosis, and reductions in Aβ levels [16,17,18]. The TNF-α blocker etanercept is being evaluated for its role in both microglial priming and system inflammation [16,19]. Additionally, BACE-1 and γ-secretase were seen as potential drug targets, although no treatments have been approved for them. One potential reason is that both have been demonstrated to be essential for functions in the body beyond Alzheimer’s disease [2,20]. 

Due to the potential impact of Alzheimer’s-related inflammation, understanding which pathways and proteins are relevant to both Alzheimer’s disease and inflammation could help identify which genes and proteins may be potential drug targets for inhibition treatments for Alzheimer’s disease. These targets offer greater potential for the development of curative drugs, as opposed to those primarily focused on symptom management. This paper firstly provides an overview of the key pathways and mechanisms of inflammation related to Alzheimer’s disease reported in the literature. Based on this review, essential genes and proteins were then identified within the explored pathways. However, not all of these genes would prove to be drug targets for treatments. Therefore, the genes and proteins linked to both Alzheimer’s disease and inflammation were extracted from the DisGeNet database. Subsequently, they underwent evaluation for their relevance to each condition through protein interaction network analysis. The ideal potential targets between both groups were then further explored to investigate their druggability (via an evaluation of existing crystal structures and clinical trials), and their possible relevance to pathways essential to functions beyond Alzheimer’s disease. 

## 2. Overview of Inflammation Pathways and Mechanisms

Inflammation in the human body is impacted by numerous complicated interconnecting pathways. Inflammation in the brain associated with the presence of Alzheimer’s disease is dominated by the classical complement pathway [14,21,22,23]. Despite Aβ’s ability to activate both the classical complement pathway and the alternative pathway [12], staining of Alzheimer’s brain tissue taken from several cortical areas in the brain, including the hippocampus, showed activation of the classical but not the alternative pathway [24]. While the many inflammation pathways form a complex network, the classical pathway seems to start the cascade [23]. Originally, the possibility of full activation of the classical complement pathway was questioned due to a lack of activation antibodies. However, it has been demonstrated that amyloid beta can bind to C1q, thereby enabling the full activation of the pathway independently of antibodies [25]. Additionally, tau proteins, which are also present in a larger amount in Alzheimer’s brains, were proven to be another antibody-independent classical activator, binding to C1q in the same antibody-independent binding site [25]. Due to their presence from the onset of the disease, Aβ and tau are both then consistently present and able to chronically stimulate the inflammation pathway [23,25]. 

**Figure 1:**
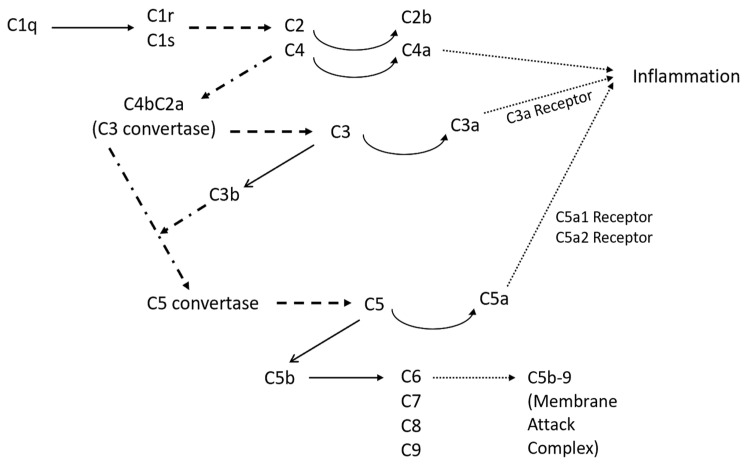
Overview of the classical complement pathway with the possible products of the pathway. The solid lines represent chemical conversion, while the dashed lines represent protease actions, and the dotted lines represent the connecting pathways that are too extensive to be incorporated in this figure.

Figure 1 shows an overview of the classical complement pathway. The pathway begins with the binding of C1q, a subcomponent of C1, and eventually leads to the formation of the membrane attack complex (C5b-9) [26]. Once C1q is bound, the serine proteases C1r and C1s automatically activate [26], and cleave C4 and C2 into two sets: larger fragments (C4b and C2a) and smaller fragments (C4a and C2b) [27]. The two larger fragments join together and cleave C3, making it the C3 convertase. Once C3 is cleaved, it forms C3a, an anaphylatoxin, and C3b, an opsonin [27]. C3b also binds with the C3 convertase to form C5 convertase, which in turn creates C5a, the most important anaphylatoxin, and C5b, which initiates the sequence of polymerization reactions and the formation of the membrane attack complex C5b-9 [28]. The membrane attack complex disrupts cell homeostasis and induces cell lysis, and therefore impacts neuronal degeneration and death. The anaphylatoxins, C3a, C4a, and C5a, interact with certain receptors, leading to a local inflammatory response, which can involve smooth muscle contraction and an increase in vascular permeability [28]. Additionally, C3a and C5a have the capacity to stimulate the expression of adhesion molecules in endothelial cells along blood vessels. Moreover, they can trigger mast cells to release mediators such as histamine and tumor necrosis factor alpha, thereby initiating the activation of the adaptive immune response [28]. C5a also increases the expression of the response factors CR1 and CR3 [28]. Genetic mutations in inflammation response factors, specifically CR1 in the classical pathway, are associated with a higher likelihood of Alzheimer’s disease [14]. Additionally, the classical complement pathway component C1q is an essential component in the activation of microglia, which directly impacts the presence of degenerative synapses [22]. Microglia, immune cells that assist in maintaining homeostasis, transition from their typical ‘resting’ form to their ‘activated’ form when disturbances to homeostasis, such as an aggregation of Aβ, induce the release of pro-inflammatory cytokines and complement proteins [29]. During periods of chronic inflammation, such as those that occur during Alzheimer’s disease, microglia maintain this activated state, which can in turn cause damage to neurons as they take on an aggressive pro-inflammatory role. This results in an elevated production of multiple cytokines with established associations to inflammation. 

## 3. Major Genes and Proteins for Alzheimer’s–Inflammation Pathways and Mechanisms

The focus of this section is to explore which pathways and mechanisms involved in inflammation may also play a role in the progression of Alzheimer’s disease based on existing research in the literature. The main pathway was found to be the classical complement pathway. While the pathways involved in Alzheimer’s disease construct a complicated network, this is the pathway that may lead to the cascade of other pathways being activated. As they have been identified as potential drug targets for other diseases and ailments, the roles of additional GPCRs relevant to Alzheimer’s disease were included, including those connected to but not directly in the classical pathway. The proteins involved in the microglial immune response were then explored due to their abnormal activities during systemic inflammation. The relevant cytokines were also described, as they play essential roles in connecting the pathways and mechanisms of Alzheimer’s inflammation. Lastly, the tangential kynurenine pathway was investigated as its activation directly contributes to the continuation and escalation of systemic inflammation. 

### 3.1. Classical Complement Pathway

**Figure 2:**
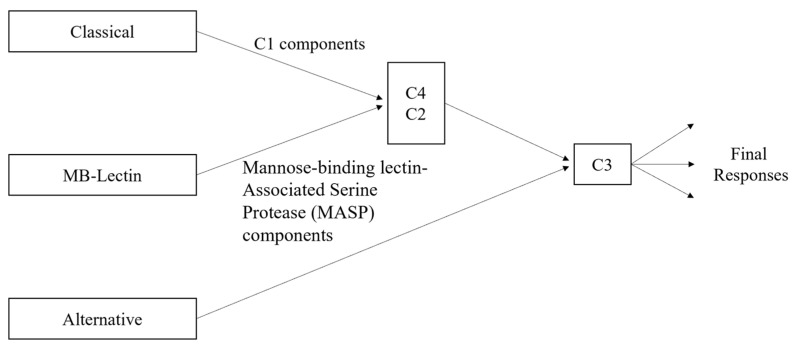
Complement pathway variations, including classical, alternative, and MB-lectin.

The main components of the classical complement pathway have different forms of impact depending on where they fall in the cascade of the pathway. Since the classical pathway is activated by the C1 complex [28], the components C1q, C1r, and C1s are essential for the initiation of the cascade. Then, if C2 and C4 are not able to be cleaved, the cascade will be halted, as their fragments form the complex C4bC2a, which is needed by the C3 convertase to continue the cascade. Additionally, C4a is one of the inflammation activators, although there is currently no known receptor for it [30]. Nonetheless, its significance lies in the fact that C4a is exclusively generated through the classical pathway, in contrast to the other anaphylatoxins, C3a and C5a, which can also originate from the alternative pathway (see Figure 2). C2b, the other cleaved component not in the C3 convertase complex, plays a role in the formation of the convertase but does not have a known role after it is released. However, it has been hypothesized that C2b may play a role in increasing vascular permeability [31]. There are three main receptors for the complement proteins involved in activating inflammation: the C3a receptor, C5a1 receptor, and C5a2 receptor [30]. All three receptors are from family A G protein-coupled receptors (GPCRs), although the actual binding mechanisms have only been successfully explored for the first two [30]. It has also been demonstrated that the C3a-derived proteins C3a7 and C3a9 can bind to FcεRI, a high-affinity IgE receptor, and that they can also inhibit mast cell signaling and cytokine secretion [30,32]. The other component cleaved from C3, i.e., C3b, binds with the C3 convertase to form C5 convertase, which is necessary to form the fragments C5a and C5b, and opsonizes fibrillar cells [12]. As previously mentioned, C5a is involved with inflammation, while C5b binds with C6, C7, C8, and several C9 components to form the membrane attack complex, C5b-9 [12]. With high levels of both Aβ and tau, and thus high levels of classical complement activation, there are then also high levels of C5b-9. The excess C5b-9 then attaches to dystrophic neurites, which are abnormal neuronal processes associated with plaques of Aβ and which have been hypothesized to be linked to the onset of dementia in Alzheimer’s patients [12,33]. Both the anaphylatoxins and opsonins (C4b, C3b, C5b) are involved with inflammation activation by signaling inflammatory cells (including microglia) that express the complement receptors, which also include C1qRp, CR1, CR3, CR4 and the previously mentioned C3a and C5a receptors [12]. Alongside the proteins directly involved in the classical complement pathway are the complement defense proteins, which are similarly upregulated with the buildup of Aβ and tau. These proteins include C4-binding proteins (C4bps), vitronectin (a multifunctional glycoprotein), and clusterin/apolipoprotein J (ApoJ) [12,34]. ApoJ is also a complement regulator alongside C1 inhibitors and CD59 [12]. It is associated with Aβ plaques and may play a role in transporting Aβ across the cerebrovasculature. The receptors involved in ApoJ transport may include gp330 but have not currently been deeply explored. The C1 inhibitor is another key player in the Alzheimer’s–inflammation pathway, as there is an imbalance of activation levels between C1 inhibitors and C1q, with Alzheimer’s brains having higher levels of C1q. CD59 plays a role in the modulation of certain forms of C5b-9, particularly those targeting homologous tissues. Its downregulation in Alzheimer’s brains has been demonstrated to potentially facilitate heightened susceptibility to increased attacks [12]. As shown in Figure 2, mannose-binding lectin (MB-lectin) plays a crucial regulatory role in the complement pathway for brain inflammation by interacting with and modulating the activities of C2 and C4. In particular, MB-lectin binding to microbial or damaged cellular surfaces triggers the activation of C2 and C4.

### 3.2. GPCRs

There are numerous GPCRs involved in the inflammation response with Alzheimer’s disease, including those outside of the classical complement pathway. GPCRs are membrane proteins that can convert extracellular signals to an intracellular response [20]. They have been shown to play a role in the hydrolytic processing of APP and thus the formation of Aβ plaques [20,35,36] by binding to β-secretase (β-site APP-cleaving enzyme 1, abbreviated as BACE-1) and γ-secretase [20,37,38,39]. Previously, BACE-1 and γ-secretase were chosen as drug targets; however, there have not been any FDA-approved treatments targeting them. It has been reported that they are involved in biological functions beyond Alzheimer’s disease and thus the negative impacts of inhibiting them are too strong [2,20]. Therefore, GPCRs may prove to be a better target for therapeutics. The occurrence of Alzheimer’s disease in the brain has been demonstrated to correlate with alterations in the expression levels of various GPCRs, including inflammation-associated receptors, hormone receptors, and neurotransmitter receptors [20]. Those that were upregulated included arginine vasopressin receptor 1a, dopamine receptor D2, metabolic glutamate receptor type 6, histamine H4 receptors, and G protein-coupled receptor 2. Those that were downregulated included cannabinoid receptor type 1, γ-aminobutyric acid receptors, 5-hydroxytryptamine receptor 1E/2A, parathyroid hormone 2 receptor, and orphan G protein-coupled receptor 22 [20,40]. In terms of BACE-1-related activity, M1 AChR [41], δ-opioid receptors (DORs) [42], A2a receptors [43], GPCR-associated sorting proteins [44], small G proteins including Rbs [42,45], and ADP-ribosylation factor 6 [46] have all been shown to play a role in regulating BACE-1 [20].

One essential subgroup of GPCRs is muscarinic acetylcholine receptors, which are synthesized by cholinergic cells and regulate multiple neuronal functions in the central nervous system (CNS) [20,47]. M1 and M3 mAChRs are associated with the activation of phospholipase C and mobilization of intracellular calcium, both of which are needed for neuronal communication and synaptic plasticity [20]. Both M1 and M3 have also been shown to increase the levels at which APP, when cleaved by α-secretase (sAPPα), releases the soluble amino terminal ectodomain [20]. sAPPα has also been shown to decrease the release of Aβ [20]. Comparatively, M2 inhibits adenylate cyclase [47], ion channels including N-methyl-D-aspartate receptors (NMDARs) [48], and calcium channels [49], decreasing levels of cyclic adenosine monophosphate (cAMP) [50] and causing other inhibitory effects [20]. mAChRs, alongside ligand-gated ion channel nicotinic acetylcholine receptors (nAChRs), are vital for the neurotransmission involved in learning and memory [20,36]. 

Another GPCR group that is involved in learning and memory is opioid receptors. The receptors and peptides in this group exist in regions of the brain that are vulnerable to Alzheimer’s disease. Additionally, it has been shown that altering the cell signaling abilities of opioid receptors impacts Aβ production and Alzheimer’s pathogenesis [20,51,52,53,54]. Within opioid receptors, the focus for investigations has been placed on the DOR, as it was shown to increase BACE-1 and γ-secretase activity [20,42]. Adenosine receptors are similarly involved in synaptic transmission regulation and neuron excitability [20]. The subtype A1 has been shown to be related to Alzheimer’s disease, as it may contribute to generating soluble APP, which mediates tau phosphorylation and has been found in dystrophic neurites and degenerating neurons near tau tangles [20,55]. The group of GPCR-regulating proteins relevant to Alzheimer’s disease can be divided into two groups: small GTPases and GPCR-associated sorting proteins (GPRASPs). Important GTPases include Rab5 and Rab7 [42], which are upregulated in Alzheimer’s disease, and Rab11 [56], which is needed for axonal sorting of BACE-1. p60TRP is a GPRASP that with increased expression can lead to the dephosphorylation of APP, inhibiting BACE-1 and reducing the APP intracellular domain [20,44].

As part of the kinin/kinin receptor system (KKS), two additional GPCRs that play essential roles in inflammation connected to Alzheimer’s disease include the bradykinin B1 receptor (B1R) and bradykinin B2 receptor (B2R) [57]. Bradykinin (BK) has been connected to the expression of IL-8 [58] and the activation of the NF-κB signaling pathway [59], both of which are connected to inflammation in the brain. Studies conducted on rats have shown that the addition of Aβ leads to increased BK levels, while increased BK levels then lead to higher levels of secreted APP β [57,60,61,62]. This cycle may contribute to neurodegeneration. Increased levels of BK have also led to tau protein phosphorylation in rat models [57,63]. B1R and B2R have also been shown to contribute to the production of inflammatory mediators. Specifically, B1R is associated with chronic inflammation, while B2R is associated with acute inflammation [57]. In mice, blocking B1R correlated with improved cognitive functions in a brain with Alzheimer’s disease [64,65]. However, evidence has been found that B1R and B2R may also take on a neuroprotective role. B1R assists in preventing leakage of the blood–brain barrier, which is an issue in Alzheimer’s disease [66,67]. When B2R is absent, memory loss and neurodegeneration are worse [68]. 

### 3.3. Microglia Involvement and Key Proteins

Microglia are immune cells that help maintain CNS homeostasis. However, their phenotypes and functionalities can change with disturbances to this homeostasis, including general aging and neurodegeneration, which can have significant impacts on the progression of Alzheimer’s disease in the brain [22]. Microglia normally exist in a ‘resting’ phenotype [69], but become activated (also known as microglial priming) when a threat to the CNS is detected, changing their morphology [22,69]. When this detected threat is prolonged, such as a chronic disease like Alzheimer’s disease, the continuous existence of microglia in the activated state may have negative consequences. The precise stimulus triggering the activation of microglia remains unknown at present. However, current hypotheses suggest potential factors, including the loss of inhibitory actions from neuronal ligands (such as CD200 and C3XCL1), the accumulation of misfolded proteins, and the phagocytosis of apoptotic bodies and neuronal debris [22]. In macrophage activation, there are two different forms: M1- and M2/M2-like [22,70]. Activation into the M1 phenotype involves the interaction of interferon-γ (INF-γ) with TLR4 signaling, and it results in increased levels of pro-inflammatory mediators and effectors. M2 polarization involves IL-4 and IL-13, and it results in increased levels of transforming growth factor-β (TGFβ), IL-10, CD163, CD206, MS4A4a/6A, and fibrinogenic and coagulation factors [22,71]. M2 is generally considered as the more anti-inflammatory phenotype [22]. 

Microglia take on the characteristics of either phenotype based on the state of both the local and systemic systems [22]. With systemic inflammation, microglia take on an aggressive pro-inflammatory phenotype, and have been shown to produce the corresponding cytokines, including IL-1, IL-6, TNF-α, TGF-β1, TGF-β2, MIP-1α, and MCP-1 [12,22,71]. Microglia, as inflammatory cells, express complement and BK receptors, which contributes to their increased presence in the vicinity of Aβ plaques [12,57]. Both microglia and astrocytes mediate Aβ and tau toxicities [2,72]. Microglia specifically have been shown to be associated with the neurons expressing Aβ with TLR2 and TLR4 in the surrounding plaques [72]. The microglia response to chronic neurodegeneration may lead to the adaptive inflammation response turning into systemic inflammation, and this exaggerated pro-inflammatory response directly contributes to neurodegenerative pathology and symptomology of Alzheimer’s disease [71]. 

The triggering receptor expressed on myeloid cells 2 (TREM2) has also been proven to play a key role in the correlation between chronic inflammation and Alzheimer’s pathogenesis [6,21,73]. Both with Aβ and tau, TREM2 has been shown to have disease stage-specific roles, as it begins in a protective position but ultimately transitions to pathogenic after some time [6]. TREM2 was shown to have an association with macrophages that come to clear Aβ as microglia diminish in their ability to remove Aβ. Based on studies with APP/PS1 and 5XFAD mice and cellular models, a lack of TREM2 was shown to reduce the amount of plaque-associated macrophages [6,73,74], which in turn worsened patients’ pathogenesis [6,75]. The macrophages and microglia without TREM2 had decreased abilities to perform their clearing duties, including the clearing of Aβ [6]. The loss of TREM2 also led to a decrease in IL-1, IL-6, and TNF-α, and astrocytosis (and thus S100β) [6]. The cell death associated with diminished TREM2 may have led to increased Aβ, which in turn caused neuronal dystrophy and death, while the remaining living cells still experienced disruption of their homeostasis [6]. Diminished levels of TREM2 were also found to increase tau insolubility and activation of neuronal stress kinases, and led to dystrophic microglia [6,76].

### 3.4. Cytokines

Numerous cytokines are involved in Alzheimer’s inflammation mechanisms. Plaque-associated cytokines, specifically IL-1, IL-6, and TNF-α, are involved in the production of C1s and C1r, but not the C1 inhibitor [12]. IL-1, involved in immunoregulation, is overexpressed early in Alzheimer’s plaque development [77,78] and its levels have been shown to directly correlate to dystrophic neurite presence [12]. IL-1 is also closely linked with Aβ precursor proteins [6,12,77,78]; it may increase production and deposition of Aβ plaques, while secreted APP in turn activates microglia and initiates overexpression of cytokines [12,14]. In addition, IL-1 can activate astrocytes, which express acute-phase and/or Aβ-binding proteins, such as α-antichymotrypsin, ApoE, and complement component C3 [12]. IL-1 overexpression also leads to S100β overexpression, which is a cytokine that promotes neurite growth and may cause the development of dystrophic neurites near Aβ plaques [12]. 

Another cytokine involved in immunoregulation and inflammation is IL-6, which specifically is involved in the growth and differentiation of central nervous system cells, and similarly has been shown to be upregulated in Alzheimer’s brains and linked with APP synthesis and expression [12]. Its production may harmfully increase through the overexpression of fellow cytokine IL-1 [23,79]. While this overexpression is harmful, IL-6 may also regulate neuronal survival and function at certain levels of exposure. However, when considered in a more holistic context, IL-6 is generally considered harmful as it is pro-inflammatory and a major pyrogen, induces acute phase proteins, and increases vascular permeability, lymphocyte activation, and antibody synthesis. This ultimately leads to CNS damage and negative behavior changes [12]. 

The mechanisms of involvement for the inflammatory cytokine TNF-α are currently disputed. Overexpression of TNF-α leads to the death of cortical neurons, but this cytokine may also play a neuroprotective role as it may induce protective molecules. Its inhibition has been shown to increase tissue damage and decrease NF-κB, which in turn produces survival factors. This dichotomy may stem from the difference in the TNF-α receptors: p55 TNF receptors are associated with cell death, and p75 TNF receptors are associated with protecting neuronal survival [12]. 

All three forms of TGF-βs (TGF-β1, TGF-β2, TGF-β3) play a role in the pathogenesis of Alzheimer’s disease and their actions are regulated via high-affinity transmembrane receptor complexes that are made of type I and II serine/threonine kinase receptor sub-units. The TGF-βs are involved in a variety of brain responses to prevent the disruption of homeostasis, including the inflammatory response, microglial activation, astrocytosis, extracellular matrix production, the accumulation and distribution of amyloid beta, the regulation of factors associated with potential Alzheimer’s disease risk (APP, ApoE, α-MAC, COX-2), and the inhibition of cell death. TGF-β1 is associated with Aβ plaques, while TGF-β2 is found with reactive astrocytes, ramified microglia, and neurons with tangles. While normally considered an anti-inflammatory cytokine, TGF-β1 is also a chemoattractant for microglia, which are pro-inflammatory, and its overexpression has been shown to increase vascular deposition of Aβ. TGF-βs may also affect COX levels, which is important as COX-2 levels are higher in tau tangles [12]. 

Another essential component of the inflammatory response is the role of chemokines [12,23]. Chemokines are a subgroup of secreted cytokines associated with migratory behavior, particularly of cells, although they recently have been shown to also be involved in haptotaxis, chemokinesis, haptokinesis, and all protective or destructive immune and inflammatory responses [80,81]. Their receptors also play a critical role in chemokine modulation and consist of cell-surface GPCRs [80]. In terms of inflammation, chemokines are essential for the mobilization of microglia [23]. One chemokine that is of particular interest to the Alzheimer’s disease and inflammation pathway is IL-8 [12,23] and its receptor CXCR2 [23]. IL-8 has been shown to have the largest increase in expression when incubated with Aβ [23,82]. CXCR2 has been shown to be associated with the neuritic plaques present in Alzheimer’s brain tissue [23,82,83]. 

### 3.5. Kynurenine and Inflammation Pathway Interactions

Involved in neuroinflammation is the Kynurenine pathway, an alternative tryptophan breakdown pathway [84,85]. This pathway is activated instead of a serotonin production path and is associated with both depression and Alzheimer’s disease. This is of particular interest because while inflammation has already been shown to directly impact Alzheimer’s, it then may also impact depression levels and progression. This in turn then impacts the progression of Alzheimer’s, as depression has been shown to worsen patient outcomes in Alzheimer’s disease [85,86]. Neuroinflammation directly impacts cognitive impairment and behavioral changes, particularly in old age, which is of concern for both clinical depression and Alzheimer’s disease [66]. Both conditions feature higher levels of pro-inflammatory cytokines, such as TNF-α and IL-6. Additionally, the inflammation present in both brains may be impacted by the deficiency of monoamines, including dopamine and serotonin [87]. Specifically, serotonin levels can be decreased when its precursor tryptophan is sent to the kynurenine pathway. This occurs when tryptophan 2,3-dioxygenase (TDO) and indoleamine 2,3-dioxygenase (IDO) are expressed, which can happen due to neuroinflammation and chronic stress [85,88,89]. IDO levels have been shown to increase with Aβ plaques and can be activated by inflammation [85,90]. Additionally, depression correlates to increased levels of IL-1 [85,91]. IL-2 may then be affected by kynurenine levels [85,92].

## 4. Discussion: Potential Drug Targets for Regulating Alzheimer’s Inflammation

**Figure 3:**
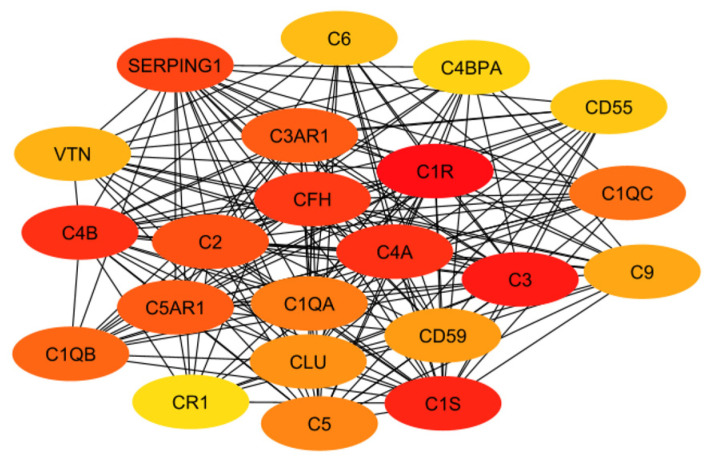
The interaction network of proteins from the classical complement pathway, with darker red and lighter yellow indicating higher and lower interactions, respectively.

**Figure 4:**
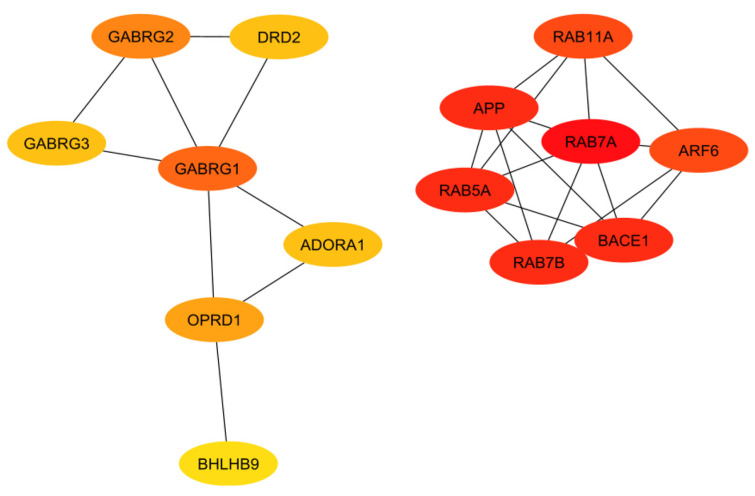
Interaction network of the GPCRs with a proven association to Alzheimer’s disease, with darker red and lighter yellow indicating higher and lower interactions, respectively.

In order to evaluate the numerous proteins mentioned in the above sections, their connections and roles in the body have to be analyzed. This work does not aim to develop new programs for drug target identification. Instead, existing tools in Systems Biology were integrated to evaluate the gene targets mentioned in the previous sections. In particular, a program named STRING, a commonly used tool in systems biology to establish the connections of each gene based on database information [93,94,95], was implemented to build a protein interaction network for genes from the classical complement pathway (Section 3.1) and GPCR genes (Section 3.2). The protein interaction networks were then imported into programs named Cytoscape [96,97] and MCODE [98] for better visualization of their highly interacting networks. The proteins were further ranked using Cytohubba [99] on the basis of their interactions, with darker red indicating the highest-ranking proteins and lighter yellow indicating low-scoring proteins. The protein interaction networks were thus built for those involved directly with the classical complement pathway (Figure 3) and GPCRs (Figure 4). The functions of the proteins shown in Figure 3 and Figure 4 are listed in Table 1 and Table 2. 

The ranking of the classical complement pathway components shows that the majority of the high-scoring genes are those in the main part of the pathway. This matches predictions, as these components are involved in starting the cascades that stem from the main pathway. These include C1R, C1s, C2, C3, and C4a/b. The lower-ranked genes seem to be binding proteins that are involved near the ends of the pathway, such as C4BPA and CR1. However, the GPCRs in Figure 4 are clustered into two distinct groups, with one group being significantly more connected than the other. This may be influenced by the fact that many of these GPCRs were identified as being independently involved with Alzheimer’s inflammation. It is important to note that the GPCRs in the right cluster relate to APP and BACE1, which, while not ideal targets themselves, are well-known in their involvement with Alzheimer’s disease. However, the RABs do not currently have full crystal structures available, which would make it hard to identify small molecule inhibitors for RABs. Additionally, they are GTPases, which exist in high quantities in the body, so targeting them could potentially be toxic or have too much of a negative impact on biological function. 

**Figure 5:**
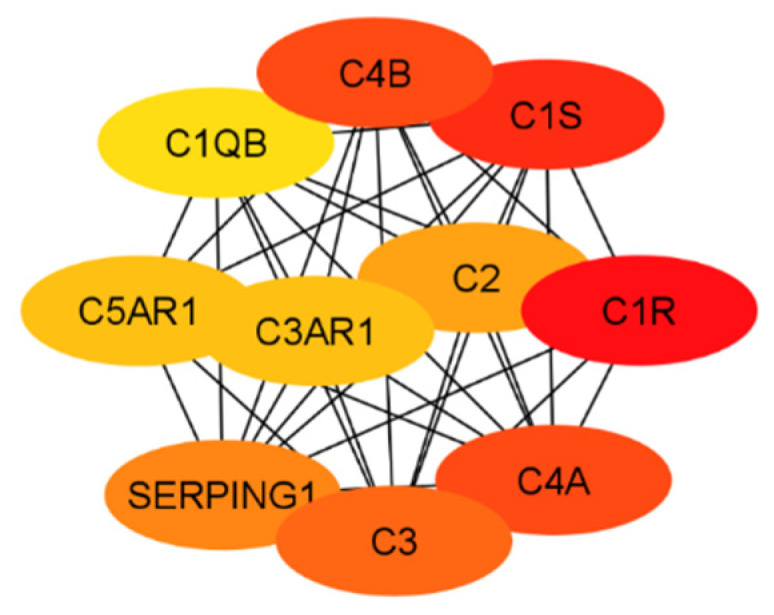
The top 10 potential targets identified from the classical complement pathway and the network of GPCRs.

The results of both the literature review and the protein interaction analysis identified numerous genes that play key roles in the systemic inflammation that arises from and exacerbates Alzheimer’s disease. However, not all of these genes would serve well as a drug target. Therefore, each gene must be considered in a larger context of the body. Some genes play too-important roles in other key bodily functions to be inhibited without serious consequences or side effects. Other targets may be acceptable in terms of function but would be difficult to target with drug therapeutics due to their location or size. Additionally, in order to be evaluated, the crystal structure of the gene must be available. To begin this evaluation, all of the proteins (encoded by genes) used in the previous evaluations (i.e., Figure 3 and Figure 4) were further evaluated together to establish the top ten total potential protein targets, depicted below in Figure 5. The crystal structures, along with clinical trials, are listed in Table 3 for these top ten potential protein targets. 

For the classical complement pathway, ideal targets would come from those that are more specific to inflammation to avoid potential side effects. Targets should not be located too early in the pathway either, as that would halt the cascade too early or in a way that negatively impacts the ability of the body to function. Many of the early components are needed to form later complexes, which in turn may play additional roles in the brain. This would also mean that a receptor would likely prove a better target than one of the mobile components. This idea also contributes to why GPCRs make ideal targets. GPCRs have already been proven to serve as drug targets for 50–60% of current drugs and work well with small molecule inhibitors [100]. However, while essential to inflammation in the brain, any cytokine discussed would not make for an ideal target, as they tend to be involved in multiple functions outside of inflammation and would also be hard to target with drug molecules. For example, IL-6 is a cytokine that may directly contribute to the negative impact of inflammation in Alzheimer’s disease. However, it is also considered a multifunctional cytokine and is responsible for multiple biological activities, including differentiating B-cells into immunoglobulin-producing cells, fever responses, neutrophil tracking, and other immune responses [101]. It also follows the kynurenine pathway. Exploring the linkage between these two pathways would help increase understanding of how inflammation works in the brain, but it would not be ideal to target this pathway directly. Although it could be investigated further due to its extreme relevance to Alzheimer’s patients, the kynurenine pathway is most relevant to depression and is not an inflammation pathway. Additionally, the ideal target must be able to interact with a potential therapy drug, which means size and accessibility must be considered, especially due to the added issue of crossing the blood–brain barrier. 

As previously mentioned, the targets from the classical component pathway would not be ideal. This would eliminate C1R/S, C4a/b, C3, and C2. SERPING1 (a C1 inhibitor) is synthesized in the liver and is significantly involved in blood coagulation, fibrinolysis, and the generation of kinins [102]. Ultimately, this would decrease its viability as a target. Additionally, the need for a crystal structure would eliminate C3AR1 and C1QB. This leaves C5AR1 as the ideal target, which will be evaluated in future work. This aligns with predictions as it is a GPCR connected to the classical complement pathway, specifically with C5a, which is an anaphylatoxin with pro-inflammatory functions. Additionally, while not entirely unstudied, not much research has been conducted evaluating antagonists that target C5AR1 while considering Alzheimer’s disease. Previous research has included peptide antagonists, which reached Phase 2 clinical trials in the treatment of psoriasis and rheumatoid arthritis [103]. However, problems arose regarding off-target activity, production costs, immunogenicity, and oral bioavailability. In 2021, for the treatment of severe active anti-neutrophil cytoplasmic autoantibody-associated vasculitis, the FDA approved avacopen (Tavneo, ChemoCentryx, South San Francisco, CA, USA), a C5a receptor [104]. Avacopen was also evaluated for patients with IgAN. Other options for targeting C5AR1 include small-molecule competitive antagonists W54011 and NDT95137327 [103]. Referring back to the top GPCRs in Figure 4, additional possible targets that have been less studied in their application for Alzheimer’s include GARBG1, which is related to the main inhibitory neurotransmitter for the CNS, gamma-aminobutyric acid (GABA) [105]. Decreased receptor levels of the A-subgroup of GABAs have been shown to have a direct relationship with CNS disorders [106]. This likely stems from the fact that GABA plays a role in stabilizing neuronal activity and initiating other essential related processes, including the formation of protective barriers (including the blood–brain barrier) [105]. The crystal structure for the predominant isoform of GABA-A receptors has also been used for numerous studies, including those that focused on the mechanisms of possible drug interactions and signaling [107,108,109,110]. Another top-scoring gene was ARF6, a small G protein [111]. ARF6 has been linked to neurodegeneration through cholesterol [112]. However, its size may prove an issue in binding with other molecules. The other GPCRs listed from the Rab family are GTPases, which exist in large quantities in the body, therefore making them a non-ideal target in terms of toxicity. 

### Limitations and Future Work

This work aims to provide a mini literature review on the genes involved in brain inflammation that may be correlated with Alzheimer’s disease progression. These genes were further evaluated using existing tools in Systems Biology to further discuss potential targets for intervening in brain inflammation for Alzheimer’s disease. Due to the space constraint, only genes, reactions, or pathways reported in the literature for their important role in Alzheimer’s inflammation were included in this work. The genes from the classical complement pathway and GPCR genes were paid more attention. These may provide new targets when compared to microglia-related genes. For example, the role of TREM2 (receptor expressed on myeloid cells 2) in chronic inflammation and Alzheimer’s pathogenesis has been extensively studied. It is not a drug target for inhibition intervention. Cytokines are too small to be a drug target. In addition, inhibiting cytokines, which are immunomodulating agents, may cause unwanted side effects. The list of drug targets discussed in this work can serve as an initial point for further exploration of targets to impede Alzheimer’s inflammation. 

For future work in investigating small molecule inhibitors for the drug targets discussed in this work, the structure of each target should be further analyzed using computational approaches, which can save time and expenditure when compared to experimental approaches. Protein–ligand docking is one of the most common approaches for drug discovery [113]. Possible docking tools would include Molsoft ICM, a top program in evaluating docking poses and predicting binding energies [114,115,116,117,118,119]. This will determine the ability of small molecules to bind with each drug target and thus allow one to determine how truly viable a target protein is. Ultimately, this would allow insight into how Alzheimer’s could be treated by targeting inflammation in the brain. In addition, certain phytochemical compounds have been shown to reduce the accumulation of amyloid beta and tau protein tangles, and some natural drug candidates are progressing into human clinical trials [120]. Phytochemical compounds can serve as a good starting point for ligand docking for screening. 

## Figures and Tables

**Table 1:** Information regarding the proteins in Figure 3.

Abbreviation	Name	Function
VTN	Vitronectin	Complement system regulator: binds C5b-7, C5b-8, C5b-9
C4B	Complement component C4b	Binds with C2A to form C3 convertase, opsonin
C1QB	B-chain of complement component C1q	C1q binds with C1r and C1s to form C1 complex, which activates classical complement pathway
SERPING1	C1 inhibitor	Inhibits C1r and C1s
C2	Complement component C2	Cleaved by C1s to form C2a and C2b
C5AR1	Complement C5a receptor 1	G protein-coupled receptor for component C5a
CR1	Complement receptor 1	Increases cleavage of C3b and C4b
C6	Complement component C6	Binds with C5b, initiating cascade that results in membrane attack complex (MAC)
C3AR1	Complement C3a receptor 1	G protein-coupled receptor for component C3a
CFH	Complement factor H	Cofactor for C3b cleavage
C1QA	A chain of complement component C1q	C1q binds with C1r and C1s to form C1 complex, which activates classical complement pathway
CLU	Clusterin	Inhibits creation of MAC
C5	Complement component C5	Cleaved by C4bC2aC3b to form C5a and C5b
C4BPA	C4-binding protein alpha	Binds to component C4b
C1R	Complement C1r subcomponent	Serine protease involved in C1 complex
C4A	Complement component C4 type A	Cleaved from C4 alongside C4B, anaphylatoxin
CD59	MAC-inhibitory protein	Inhibits creation of MAC
C1S	Complement C1s subcomponent	Serine protease involved in C1 complex
C3	Complement component C3	Cleaved by C4bC2a to form C3a and C3b
CD55	Complement decay-accelerating factor	Destabilized C3 and C5 convertases
C1QC	C chain of complement component C1q	C1q binds with C1r and C1s to form C1 complex, which activates classical complement pathway
C9	Complement component C9	Binds with C5b-8 to form MAC

**Table 2:** Information regarding the proteins in Figure 4.

Abbreviation	Name	Function
GABRG1	Gamma-aminobutyric acid receptor subunit gamma-1	GPCR that inhibits neurotransmission and is involved in several signaling pathways
GABRG2	Gamma-aminobutyric acid receptor subunit gamma-2	GPCR involved in inhibiting neurotransmission, major component of GABA-A receptors
GABRG3	Gamma-aminobutyric acid receptor subunit gamma-3	GPCR involved in inhibiting neurotransmission, major component of GABA-A receptors
DRD2	Dopamine receptor D2	GPCR that inhibits adenylyl cyclase activity
OPRD1	Opioid receptor delta 1	Involved in opioid and enkephalin receptor activity and response
BHLHB8	Basic helix-loop-helix domain class B, 8	Essential for glucose homeostasis and calcium ion transmembrane transportation
APP	Amyloid beta precursor protein	Leads to generation of Aβ
RAB5A	Ras-related protein Rab-5A	Small GTPase involved in regulation of intracellular membrane transportation
RAB11A	Ras-related protein Rab-11A	Small GTPase involved in regulation of secretion and intracellular membrane transportation
RAB7A	Ras-related protein Rab-7A	Small GTPase involved in regulation of intracellular membrane transportation
RAB7B	Ras-related protein Rab-7B	Small GTPase involved in regulation of intracellular membrane transportation and protein degradation
ARF6	ADP-ribosylation factor 6	Binding protein involved in membrane transportation
BACE1	β-site APP-cleaving enzyme 1	Limits Aβ generation in the brain

**Table 3:** Top 10 potential targets.

Target	Crystal Structure	Clinical Trials
C1R	Yes	Conestat alfa, Human C1-esterase inhibitor, Palivizumab, Zinc acetate, Zinc cation
C1S	Yes	Sutimlimab, Conestat alfa, Copper, Human C1-esterase inhibitor, Zinc acetate
C4A	Yes	Human immunoglobulin G
C4B	Yes	Zinc cation, Copper, Human immunoglobulin G, Zinc acetate, Zinc chloride
C3	Yes	Clozapine, Pegcetacoplan, Copper, Human Immunoglobulin G, Zinc acetate
SERPING1	Yes	Copper, PPL-100, CINRYZE, RHUCIN
C2	Not whole	Abaloparatide
C5AR1	Yes	Avacopan, PMX 205, Calcium, COMPSTATIN
C3AR1	No	Histamine, D-Threonine, D-Tyrosine, Serine, Calcium
C1QB	No	Bevacizumab, Cetuximab, Etanercept, Palivizumab, Zinc acetate

## Data Availability

Data may be provided upon request.
